# Sensitivity and specificity of a brief scale to evaluate psychological violence at work in Peruvian health professionals

**DOI:** 10.1186/s13104-022-05959-8

**Published:** 2022-02-16

**Authors:** Luis Fidel Abregú Tueros, Roger dos Santos Rosa

**Affiliations:** Grupo de Investigación en Salud en el Trabajo, Universidad Nacional Agraria de la Selva, Huánuco, Tingo María, Perú

**Keywords:** Workplace violence, Utility of scales, ROC curve, Cut-off point, Health professionals, Health care workers

## Abstract

**Objective:**

Most studies in Latin America that evaluate psychological violence at work (PVW) focus on measuring occurrences of PVW. However, the discriminative validity and randomness of instruments used for evaluating incidents of PVW that are generated by agents internal to the workplace in the Peruvian health sector have not yet been studied. The objective of this study was to determine the sensitivity and specificity of the Scale of Psychological Violence in Health Professionals (SVP-Health) in the Peruvian population. For this purpose, a cross-sectional study based on the two-stage administration of guided surveys and ROC (receiver operating characteristic) curve analysis was performed.

**Results:**

The study included 188 professionals from ten care centres in Peru. The professionals were divided into two groups of 94 subjects: those who had experienced PVW and those who had not. The average age of the participants was 36.8 ± 10.5 years; their work experience ranged between one and 35 years; and 59% of the sample were women. According to an analysis based on the ROC curve, (a) there was a significant area under the curve (AUC = 0.899) with adequate randomness; and (b) the SPV-Health adequately distinguished subjects with PVW from those without PVW (89% versus 94%).

**Supplementary Information:**

The online version contains supplementary material available at 10.1186/s13104-022-05959-8.

## Introduction

Psychological violence at work (PVW) refers to an intentional action in which workers are verbally assaulted, threatened and/or humiliated during their professional practice [1, 2] in ways that do not involve physical violence [3]. PVW is exacerbated in the health sector due to the precariousness of working conditions, which commonly involve psychosocial risk factors [4–6]. There are no differences in the rates of PVW between developed and underdeveloped countries, between health groups, or between stable and temporary work conditions [7–10]; however, at the individual level, the effects of PVW can vary according to the individual’s resilience [6, 11].

At the global level, there is a considerable variety of instruments available for evaluating and monitoring the prevalence and impact of PVW [3, 10], but few studies have examined Peruvian health professionals’ experiences with PVW perpetrated by internal agents (that is, superiors or colleagues) [9, 12] and PVW related to interpersonal conflicts or demands. This is partly because some analytical tools, for example, structured formats or the aggressive behaviour scale [10, 13, 14], have been validated for Spain, Mexico, Colombia, Ecuador, Chile and Bolivia but not for the Peru.

Specifically, in Bolivia, Ecuador and Chile, although the convergent validation of the General Health Questionnaire (12 items) has been established and diagnostic scales have been created to measure violence and psychological harassment (including external violence) in workers in the service, industry, commerce and education sectors, the sensitivity and specificity of these diagnostic scales have not been determined [15, 16].

The instruments used in Peru to evaluate PVW in different health care settings have limitations, since their usefulness and the classifications they propose have not been established and, consequently, they do not meet the standards of use for psychological tests [17, 18].

Due to the limitations of the existing measures, it is necessary to develop an instrument with sufficient utility, discriminative capacity and randomness to classify workers exposed to PVW [19]. In addition, it is necessary to standardize the criteria for the interpretation of the results to identify, evaluate and compare the prevalence of PVW according to location (Lima versus other cities), work activity (nursing, medicine and others) and employment status (stable versus temporary) [12, 17] to verify corrective actions and establish baselines in matters of mental health at work. The lack of tools with reference scales and discriminative capacity hinders standardized evaluations of PVW.

This report complements the Scale of Psychological Violence in Health Professionals (SPV-Health) assessment and allows us to answer the following question: What is the best cut-off point for classifying cases with and without psychological violence according to the SPV-Health? Our objective is to determine the sensitivity and specificity of the SPV-Health in the Peruvian population.

## Main text

### Methods

This is a cross-sectional study based on the administration of guided surveys in two stages: (a) the classification of groups of equal size comprised of participants with and without PVW according to the external criterion of job satisfaction (JS), which was evaluated using an overall job satisfaction scale (OJS); and b) evaluation of the sensitivity and specificity of the SPV-Health in both groups (participants with and without PVW). The surveys were conducted between December 2019 and February 2020. To determine the sample size, the minimum recommendations established for external validation and receiver operating characteristic (ROC) curves were adopted: groups of n ≥ 176–200 participants [20, 21] with similar sex distributions [12]. No calculation was required.

The inclusion criteria for the participants were (a) complete responses to both assessment instruments (the OJS and SPV-Health) and (b) having worked at least 6 months before the time of the evaluation. The exclusion criterion was less than 2 years of work experience [12].

### Instruments

#### SPV-Health (short scale used to evaluate psychological violence in health professionals)

For this study, a scale was validated that comprises 22 items referring to three types of violence in the context of Peruvian health care settings: isolation, intimidation and discreditation [22]. Each item is rated on a 4-point Likert scale (ranging from 1, “never” to 4, “always”) (see Additional file 1). The scale showed good reliability and validity in a population of 316 health professionals [22]. Its properties are as follows:

- Good content validity, evaluated with the judgement of five experts (doctors, nurses and teaching psychologists), with a Kendall coefficient (W = 0.509; p < 0.026) that exceeded the established minimum values [23].
- Appropriate construct validity, with factors that explain up to 54.3% of the total variance, which exceeds the minimum (50%) [24]; confirmatory factors of moderate adjustment; root mean square error of approximation and standardized residual of the mean square root above the acceptability limit; and acceptable comparative fit and Tucker–Lewis indices (0.60).
- Good convergent validity with the OJS (r = − 0.769; p < 0.0003).
- Good internal consistency reliability, with a global Cronbach’s alpha coefficient of α = 0.803 and an item Cronbach’s alpha of α = 0.800 (acceptable values are α = 0.70–0.80) [18]. Reliability was assessed in terms of response stability; there were significant differences between the first and second applications, which were separated by a 4-month interval (p < 0.008).

#### Overall Job Satisfaction Scale (OJS)

The OJS consists of 15 items grouped into two subscales: (a) intrinsic job satisfaction, which is related to task-based factors such as recognition, responsibility and promotion (7 items); and (b) extrinsic satisfaction, which is related to satisfaction with the organization, schedules, wages and physical conditions of the work (8 items). Each item is rated on a 7-point Likert scale (1, “very dissatisfied” to 7, “very satisfied”). The scale presented good reliability and validity in 518 Spanish nurses [14], with an overall reliability of alpha 0.75. Its construct reflects experiences and emotional responses at work [15].

### Procedure

The study subjects were informed about the objectives of the research, and their voluntary participation was recorded through a letter of consent in which the participants’ anonymity and the confidentiality of the data were guaranteed. The simultaneous collection of data was carried out by organizational psychologists and health professionals.

### Statistical analysis

To evaluate the sensitivity and specificity of the SPV-Health in groups of participants with and without PVW, a ROC curve analysis was performed, and the area under the curve (AUC), the standard error (SE) and the cut-off point that maximizes the sensitivity–specificity of the instrument were determined [20, 25]. To divide the results into three levels, the variables JS and PVW were scaled based on enneatypes; for example, enneatypes 1 to 3 corresponded to a low level of JS and a maximum score of 66 points on the OJS (Additional file 2). To confirm the agreement between PVW and JS, Cohen’s kappa coefficient was calculated. The data were processed in Stata v15.

The datasets generated and analysed during the current study [26] are available in the [Figshare] repository, [Persistent web link to datasets], DOI [https://doi.org/10.6084/m9.figshare.14308937.v1].

### Ethics

The study was approved according to Resolution 292/2018-D-FCEA of Universidad Nacional Agraria de la Selva (Peru). Informed consent was obtained from all study participants, respecting their privacy and free will.

### Results

We simultaneously used the OJS and the SPV-Health to evaluate 188 health professionals from 10 health centers in different Peruvian cities. Of these professionals, 94 had experienced PVW, and another 94 had not (Table 1). The samples were homogenous in terms of gender and work activity according to the relevant tests (p < 0.163; p < 0.024), thus fulfilling the conditions indicated above [12]. The average age of the participants was 36.8 ± 10.5 years, and the predominant occupational group was health care providers (80.3% of the total), of whom 18.6% worked in emergency services (Table 1).

**Table 1:** Social and labour characteristics of the population under study

Feature	Group with PVW (n = 94)	Group without PVW (n = 94)	Total (%) n = 188	p*
Origin				
City of Lima	81	53	134 (71.3)	0.0004
Other cities	13	41	54 (28.7)	
Activities				
Nursing	53	37	90 (47.9)	0.024
Medicine	11	8	19 (10.1)	
Other professions	16	19	35 (18.6)	
Management	14	30	44 (23.4)	
Employment status				
Stable	57	57	114 (60.6)	0.0002^a^
Temporary	37	37	74 (39.4)	
Sex				
Male	44	33	77 (41.0)	0.163
Female	50	61	111 (59.0)	

^a^Calculated for the total frequency

*X^2^ test for p < 0.05

#### Sensitivity and specificity

**Fig. 1:**
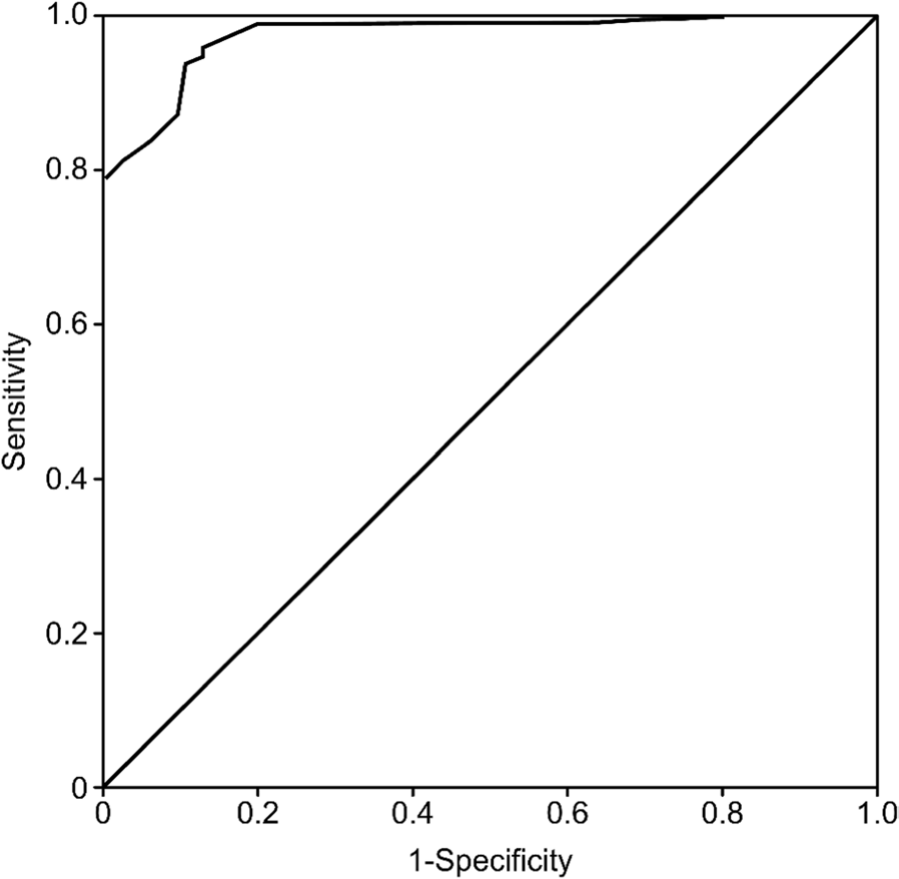
ROC curve for psychological violence at work evaluated using the SPV-Health scale

The results showed a statistically significant ROC curve, with an AUC = 0.899 (higher than the suggested minimum of AUC = 0.70) [20, 25] and a standard error (SE) = 0.02, p < 0.01 (95% CI 0.855–0.942), demonstrating that the individuals could be identified randomly using the SPV-Health scale. The scale had a good capacity to classify those who have and those who have not experienced PVW: 89% versus 94% of cases, respectively (Fig. 1). The cut-off point that maximized sensitivity (S = 0.94) and specificity (E = 0.89) was 35 out of a maximum score of 73 on the scale, which is equivalent to good predictive coordinates (ROC curve: ≥ 0.80) [25]. The maximum Youden index (J = 0.83) indicated an adequate predictive limit and discriminative capacity [20].

#### Job satisfaction as an external criterion

The magnitude of concordance or reproducibility between two categories of JS and levels of PVW among health professionals was good (k = 0.766), given that k = 0.61–0.80 is adequate (Table 2). When groupings of three categories were performed for PVW and JS, the agreement was also significant according to both the chi-square test and Fisher’s exact test (Additional file 3).

**Table 2:** Concordance between psychological violence at work and job satisfaction

	Job satisfaction (JS)		Total n = 188	Cohen’s kappa ^index^	Standard error	p
	Group with low JS (n = 94)	Group with high JS (n = 94)				
Psychological violence at work (PVW)						
With PVW	11 (11.7%)	83 (88.3%)	94	0.766	0.047	0.001
Without PVW	83 (88.3%)	11 (11.7%)	94			

p = bilateral significance for p < 0.05

Although the normal distribution of scalar scores was limited, the degree of nonparametric negative correlation between PVW and JS was high (ρ = − 0.850; p < 0.0001), which confirms that when the PVW levels increase, the perception of JS decreases, and vice versa. Up to 81.7% of the changes in JS were explained by PVW. The JS groups differed in terms of the socio-occupational characteristics of “origin” and “activities” but were similar in terms of “employment status” and “sex” (Additional file 4).

### Discussion

This study confirms that the SPV-Health scale shows good randomness for classifying individuals with and without PVW and establishes the cut-off point that maximizes the scale’s sensitivity and specificity (89% and 94%, respectively). We also found that PVW explains up to 81.7% of the changes in the criterion factors of extrinsic and intrinsic JS.

Taking into account the evidence gaps described above, which are related to the usefulness of the instruments for evaluating PVW in the health sector (a specific and contextual area [12]), we highlight three indirectly binding aspects:

- The SPV-Health, which evaluates violence by agents internal to the workplace [1–3], differs from the inventory of Diaz et al. [16] in terms of the cut-off scores used to determine high levels of violence and psychological harassment (45 in the latter versus 35 in the SPV-Health); another difference is that the SPV-Health encompasses physical and verbal violence generated by internal and external agents [16]. In theory, these represent hierarchical violence [2, 3] and horizontal violence (Type III) and external violence (Type II) [12].
- The ROC curve results in other areas [3, 19] provide conclusive support for the SPV-Health due to its discriminative capacity (AUC = 0.89 versus the limit of AUC > 0.80) [19] and its randomness of selection, which are adequate in both cases [3, 19].
- Regarding the Pakistani stress questionnaire (AUC = 0.64; kappa = 0.84 [6] versus AUC = 0.89; k = − 0.766 in the present study), the value of the SPV-Health is reinforced in clinical and legal environments, given the convergence of stress as an underlying factor [6]. A decrease in JS negatively affects organizational commitment [5] and increases the levels of stress and exhaustion in health workers [2, 10], and PVW becomes a reason for leaving the job [12].

To the best of our knowledge, this is the first study to evaluate the practical application of and prospects for using the SPV-Health, which uses uniform criteria to classify PVW [3, 4] and makes it possible to collect baseline and follow-up responses within the framework of clinical psychology and organizational psychology [3, 6].

### Conclusion

The ROC curve analysis indicates the good randomness of the SPV-Health instrument and establishes that the cut-off point for the scale’s maximum sensitivity and specificity is 35 (out of a maximum score of 73). The scale can be used in different health centres in Peru. The SPV-Health exhibits a good ability to discriminate between individuals with PVW and those without PVW (detecting 89% of those with PVW and 94% of those without PVW).

## Limitations

The limitations of this study are related to the low level of quality control in the interviewers’ application of the instrument. The AUC estimation was performed with 94% of the required clinical sample size (n = 188/200) [12]. However, as our comparison groups had the same sample sizes, the coordinates of the determined ROC curve maintained their current positions [12, 22], which increases the usefulness of the SPV-Health scale. The SPV-Health construct validation did not include confirmatory factor analysis given the absence of an additional evaluation sample [22]. Future studies may address the interaction of PWV and resilience and perform stratified analysis.

## Supplementary Information

**Additional file 1: SPV-Health.** A brief Scale to Assess Psychological Violence in Health Professionals that provides socio-occupational information and 22 anonymous responses on psychological violence that occurred during the last 6 months.

**Additional file 2: Scales for the division of variables.** The table shows the “enneatypes” (1–9) that were used to divide the “JS” and the “PVW” variables into three levels. For the lowest level, the base scores of the “OJS” scale are located within Enneatypes 1–3. In the second and sixth columns, the ranges and frequency distribution coefficients of the scores for each enneatype are shown.

**Additional file 3: Frequency agreement according to “PVW” and “JS” levels.** The table shows the degree of agreement according to three levels (low, medium, high) evaluated by two tests (chi-square and Fisher’s exact test) and the corresponding relative frequencies.

**Additional file 4: Job satisfaction according to socio-labour characteristics.** The results for the comparison of “JS” are presented according to the number of cases in each of two groups for “origin”, “employment status” and “sex” and in each of four groups for “activities”.

## Data Availability

The dataset used and/or analysed during the study is available at the following link: [Figshare]. They can also be obtained from the authors of this article [26].

## Abbreviations

SPV-Health: Scale for Assessing Psychological Violence in Health care
PVW: Psychological violence at work
ROC: Receiver operating characteristic
AUC: Area under the curve
SE: Standard error
S: Sensitivity
E: Specificity
JS: Job satisfaction
OJS: Overall Job Satisfaction Scale
