# Supplementary material for: Sensitivity and specificity of a brief scale to evaluate psychological violence at work in Peruvian health professionals
Source: BMC Res Notes. 2022 Feb 16;15:62. doi: 10.1186/s13104-022-05959-8 (PMC8848785; doi:10.1186/s13104-022-05959-8)
Supplement: Supplementary file 1 — Additional file 1: SPV-Health. A brief Scale to Assess Psychological Violence in Health Professionals that provides socio-occupational information and 22 anonymous responses on psychological violence that occurred during the last 6 months. [file 13104_2022_5959_MOESM1_ESM.pdf]

Anonymous
SPV-Health (a short scale to assess psychological violence in health professionals)

Code	Health Centre	Profession	Work Area	length of service (years)	Age (years)	employment status	Sex

Kindly mark with an "X" the answer option that best suits you.

N°	Items	Never	Occasionally	Most often	Always
	**In the last six months**				
1	You are penalised even for minor faults				
2	Your training or capacity building opportunities are blocked				
3	You're mocked, slandered/publicly defamed				
4	You're rejected by avoiding eye contact and belittling gestures.				
5	You're hindered from communicating with colleagues or others.				
6	Your personal reputation is attacked by devaluing your work.				
7	You're entrusted with tasks of higher competences				
8	You are accused of (manipulated) negligence.				
9	Your work is criticised or you are verbally attacked.				
10	You're assigned tasks with very short deadlines				
11	You're excluded from meetings where decisions are taken				
12	You're prevented from accessing important information				
13	Spread malicious rumours about you				
14	Your work is devalued				
15	Your presence is ignored				
16	New tasks are assigned to you without a break				
17	Your employment successes are ignored				
18	You're interrupted when you speak				
19	You're not allowed to express yourself				
20	Opportunities for promotion and better working conditions are blocked.				
21	Your reputation or personal image is attacked				
22	Attempt to humiliate or deride you				

Thank you very much for your kind reply!
