# Supplementary material for: Sensitivity and specificity of a brief scale to evaluate psychological violence at work in Peruvian health professionals
Source: BMC Res Notes. 2022 Feb 16;15:62. doi: 10.1186/s13104-022-05959-8 (PMC8848785; doi:10.1186/s13104-022-05959-8)
Supplement: Supplementary file 2 — Additional file 2: Scales for the division of variables. The table shows the “enneatypes” (1–9) that were used to divide the “JS” and the “PVW” variables into three levels. For the lowest level, the base scores of the “OJS” scale are located within Enneatypes 1–3. In the second and sixth columns, the ranges and frequency distribution coefficients of the scores for each enneatype are shown. [file 13104_2022_5959_MOESM2_ESM.pdf]

Additional file 2. Scales based on "enneatypes" were used to distinguish levels of job satisfaction and psychological violence at work

Job satisfaction (JS)				Psychological violence at work (PVW)			
Enneatypes	Maximum frequency (Rx = 48)	Score	Level	Enneatypes	Maximum frequency (Rx = 51)	Score	Level
9	2 (1.9)	102-103	High (93-103)	9	2 (2.0)	71-72	High (61-72)
8	3 (3.4)	99-101		8	4 (3.6)	67-70	
7	6 (5.8)	93-98		7	6 (6.1)	61-66	
6	8 (8.2)	85-92	Medium (67-92)	6	9 (8.7)	52-60	Medium (34-60)
5	10 (9.6)	75-84		5	10 (10.2)	43-51	
4	8 (8.2)	67-74		4	9 (8.7)	34-42	
3 [a]	6 (5.8)	61-66 [a]	Low (56-66)	3 [b]	6 (6.1)	28-33 [b]	Low (22-33)
2	3 (3.4)	58-60		2	4 (3.6)	24-27	
1	2 (1.9)	56-57		1	2 (2.0)	22-23	

Rx = Range

[a] Cut-off point for low "JS": 66 points (OJS scale).

[b] Cut-off point for low "PVW": 33 points (PVS-Health scale).
