# Supplementary material for: Sensitivity and specificity of a brief scale to evaluate psychological violence at work in Peruvian health professionals
Source: BMC Res Notes. 2022 Feb 16;15:62. doi: 10.1186/s13104-022-05959-8 (PMC8848785; doi:10.1186/s13104-022-05959-8)
Supplement: Supplementary file 3 — Additional file 3: Frequency agreement according to “PVW” and “JS” levels. The table shows the degree of agreement according to three levels (low, medium, high) evaluated by two tests (chi-square and Fisher’s exact test) and the corresponding relative frequencies. [file 13104_2022_5959_MOESM3_ESM.pdf]

**Additional file 3.** Concordance between levels of psychological violence at work and levels of job satisfaction

| | | Level of job satisfaction (JS) | | | Total | Chi-square test | df | p |
|---|---|---|---|---|---|---|---|---|
| | | Low | Medium | High | | | | |
| Level of psychological violence at work (PVW) | Low | 0 | 9 | 78 | 87 | 231.36 | 4 | 0.0001 |
| | Medium | 4 | 90 | 1 | 95 | | | |
| | High | 5 | 1 | 0 | 6 | | | |
| Total (%) | | 9 (4.8) | 100 (53.2) | 79 (42.0) | 188 (100.0) | | | |

Note: Fisher's exact test = $p < 0.0001$.

p = bilateral significance for $p < 0.05$.
