# Supplementary material for: Sensitivity and specificity of a brief scale to evaluate psychological violence at work in Peruvian health professionals
Source: BMC Res Notes. 2022 Feb 16;15:62. doi: 10.1186/s13104-022-05959-8 (PMC8848785; doi:10.1186/s13104-022-05959-8)
Supplement: Supplementary file 4 — Additional file 4: Job satisfaction according to socio-labour characteristics. The results for the comparison of “JS” are presented according to the number of cases in each of two groups for “origin”, “employment status” and “sex” and in each of four groups for “activities”. [file 13104_2022_5959_MOESM4_ESM.pdf]

Additional file 4. Comparison of job satisfaction according to the socio-occupational characteristics of Groups 2 (Origin, Employment status, Sex) and 4 (Activities)

Feature	Obs	Rank sum	Expected	Mann-Whitney test	p
Origin					
City of Lima	134	11331	12663	-4.481	0.0001
Other cities	54	6435	5103		
Employment status					
Stable	114	11199.5	10773	1.329	0.1839
Temporary	74	6566.5	6993		
Sex					
Male	77	6725.5	7276.5	-1.706	0.0881
Female	111	11040.5	10489.5		
Activities					
Nurcing	90	7569.0 [a]	H = 9.209 [a]	gl =3 [a]	0.0266 [a]
Medicine	19	1827.0			
Other professions	35	3335.5			
Management	44	5034.5			

[a] According to Kruskal-Wallis test (H).

p = bilateral significance for $p < 0.05$.
